# Random Frequency Division Multiplexing

**DOI:** 10.3390/e27010009

**Published:** 2024-12-27

**Authors:** Chanzi Liu, Jianjian Wu, Qingfeng Zhou

**Affiliations:** The School of Electric Engineering and Intelligentization, Dongguan University of Technology, Dongguan 523808, China; liucz@dgut.edu.cn (C.L.); wjj9302@gmail.com (J.W.)

**Keywords:** RFDM, random matrix, DNN, MIMO

## Abstract

In this paper, we propose a random frequency division multiplexing (RFDM) method for multicarrier modulation in mobile time-varying channels. Inspired by compressed sensing (CS) technology which use a sensing matrix (with far fewer rows than columns) to sample and compress the original sparse signal simultaneously, while there are many reconstruction algorithms that can recover the original high-dimensional signal from a small number of measurements at the receiver. The approach choose the classic sensing matrix of CS–Gaussian random matrix to compress the signal. However, the signal is not sparse which makes the reconstruction algorithms ineffective. We take full account of the great power of deep neural networks (DNN) to detect the signal as it is an underdetermined equation. The proposed RFDM establishes a novel signal modulation and detection method to target better transmission efficiency, and the simulation results show that the proposed method can achieve good BER, offering a new research paradigm to improve the spectrum efficiency of a multi-subcarrier, multi-antenna, multi-user system.

## 1. Introduction

Next-generation wireless systems and standards, beyond 5G and 6G, are expected to support a variety of services, such as vehicle-to-everything communications, autonomous driving, remote surgery, aerial vehicles, and operations in extremely high-frequency bands. However, the spectrum resource limitations, high propagation loss, complex network architectures, and energy consumption issues we face all require new modulation techniques and waveforms, which are able to cope with various challenging requirements and show robustness in high mobility scenarios.

Current systems are based on Orthogonal Frequency Division Multiplexing (OFDM) which can achieve near-optimal performance in time-invariant frequency selective channels. Nevertheless, due to its high PAPR (Peak-to-Average Power Ratio) and large Doppler frequency shifts, its performance drastically decreases by inter-carrier interference (ICI). To address this issue, Orthogonal Time Frequency Space (OTFS) is designed to multiplex information symbols in the delay-Doppler (DD) domain, effectively mitigating the effects of channel delays and Doppler shifts [1]. The affine frequency division multiplexing (AFDM) employs the inverse discrete affine Fourier transform to modulate symbols into a “warped” time-frequency domain to handle channel delays and Doppler shifts [2]. The interleave frequency division multiplexing (IFDM) proposed the interleave and Fourier transform to enhance the channels’ statistical stability, and the low-complexity detectors are able to ensure bit error rate (BER) performance [3].

The core of OFDM, OTFS, AFDM, and IFDM lies in constructing sparse equivalent channel matrices that facilitate low-complexity signal detection algorithms, balancing optimal performance with implementation complexity. While the maximum likelihood (ML) detector is optimal, its exhaustive search process makes it inefficient for multiple-input–multiple-output (MIMO) systems [4]. Although the zero-forcing (ZF) and minimum mean square error (MMSE) detectors which are widely used in MIMO systems provide suboptimal performance compared to ML; these detectors require the computation of the inverse of the Gram matrix of the channel. When the channel matrix has a large dimension, it will make these detectors impractical due to their high computational complexity, which poses challenges for the system’s hardware efficiency and overall cost. Many other low-complexity detectors have been investigated. Paper [5] proposed a highly efficient detection technique achieved by dimensionality reduction in the channel matrix and the BER performance is comparable to the MMSE detector. Paper [6] presented a suboptimal ML detector which is suitable for the flexible implementation of OFDM-IM (index modulation) systems.

However, the new features of future communications [7], such as complex scenarios with unknown channel models, high speed, and accurate processing requirements, make traditional methods no longer suitable; embedding deep learning (DL) theories into communication systems has attracted a lot of attentions, and researchers believe that DL can achieve further performance improvements in complex scenarios for the following reasons: firstly, the deep network has been proven to be a universal function approximator with superior algorithmic learning ability. Secondly, handling large data is an essential feature of DL because of the instinctive nature of its distributed and parallel computing architectures. Finally, DL-based communication systems can break the artificial block structure to achieve global performance improvement because they are trained to optimize end-to-end performance.

There has been a lot of research on combining DL into MIMO communication, such as channel estimation, signal transmission, detection techniques, and so on. For example, Sun proposed a novel learn iterative search algorithm (LISA) for signal detection in a MIMO system with the DNN. Through training, optimal parameters of the neural networks are learned and thus near ML detection performance is obtained in both fixed and varying channel models under quadrature phase shift keying (QPSK) modulation than classical detectors [8]. A DL-aided Logarithmic Likelihood Ratio (LLR) correction method is proposed for improving the performance of MIMO receivers [9], where it is typical to adopt reduced-complexity algorithms for avoiding the excessive complexity of optimal full-search algorithms. A DNN is trained for detecting and correcting both over-confident and under-confident LLRs with a relatively low complexity compared to the popular reduced-complexity receiver detector techniques.

This paper investigates a RFDM algorithm for faster transmission efficiency. It compresses all symbols by a rectangular matrix. This matrix is the measurement matrix of CS, which can compress and measure signals simultaneously. Of course, the measurement matrix needs to satisfy certain conditions, and there are many matrices that meet these conditions; the Gaussian random matrix is one of the most commonly used measurement matrices which can ensure the reconstruction of the original high-dimensional signal with high probability. Meanwhile, since the signal is not a sparse signal, which is the premise of CS, the reconstruction algorithm cannot detect the signal from a small number of measured values. A DNN is leveraged to learn the implicit mapping function between the received compressed signals and original transmitted signal. The major contributions of this paper can be summarized as follows: (1) The investigated RFDM scheme compresses the original high-dimensional signal through a Gaussian random matrix, which can transmit more data, or the same amount of data, using fewer subcarriers or antennas. (2) A DNN detector is proposed to recover the original high-dimensional signal directly from the low-dimensional data by using the pre-trained DNN network. This novel signal multiplexing and detection method for MIMO communication systems is useful when the number of antennas or subcarriers is limited. That is to say, the main advantage is that the amount of data to be transmitted is reduced, or that the same subcarrier or transceiver antenna can transmit more data. The compression rate of our experimental setting is the fixed 0.75 which is related to the properties of measurement matrix and the accuracy of the reconstruction algorithm.

The remainder of this paper is organized as follows. The basic concept of OFDM and the origin of our idea are introduced in Section 2; this will help us to understand the feasibility of our solution. Section 3 describes the DNN-based RFDM algorithm which consists of two parts: the compression of the transmitted signal and the reconstruction of the signal based on DNN model. The schemes extending to multiple antennas are described in Section 4. The simulation results are analyzed in Section 5. Finally, the paper is concluded in Section 6.

## 2. Preliminaries

### 2.1. OFDM Modulation

A symbol vector $s\in C^{N\times 1}$ in the frequency domain is modulated by the inverse fast fourier transform (IFFT) to generate a time-domain signal x,

(1) $$x=F_{N}^{H}s,$$

where $F_{N}$ denotes the *N*-point normalized FFT matrix and $F_{N}^{H}$ is its conjugate transpose matrix. Then, a cyclic prefix (CP) is added to x and transmitted over mobile time-varying channels. After OFDM demodulation, the received signal is

(2) $$y=F_{N}\left(Hx+v\right),$$

where H is a circulant channel matrix and $v∼\mathrm{CN}(0,\sigma^{2}I)$ is additive white Gaussian noise (AWGN).

### 2.2. Inspirations and Beginnings

CS is a signal processing technique that reconstructs a signal from a small number of measurements by exploiting its sparsity [10]. It relies on three principles: (1) the signal $s\in C^{N\times 1}$ is sparse or can be represented with fewer non-zero coefficients in some specified domains, with at most *K* non-zero components. (2) This sparse or approximately sparse signal can be undersampled by a matrix A with specific properties. Since it projects the signal in a low-dimensional subspace spanned by the rows of the sensing matrix A, A is either randomly generated or designed based on the restricted isometry property (RIP) or its incoherence with a representation basis [11]. Much effort has been made in the signal processing community to design A such that the structure of the data is preserved in the low-dimensional subspace. There are many matrices which satisfy the RIP, such as Gaussian random matrices, Bernoulli random matrices, and so on. (3) The reconstruction algorithm can uniquely solve the underdetermined equation by minimizing the following $\ell_{0}$-norm:

$${\min∥s∥}_{0}\;s.t.\;z=As,$$

where z is the received signal [12]. The nature of CS is to recover the original high-dimensional signal from a small number of measurements, inspired by CS principle; it is natural to apply this idea to the communication system [13]. Then, we propose the RFDM scheme. At the transmitter, the signal is compressed to lower dimensions and can be reconstructed at the receiver.

## 3. Random Frequency Division Multiplexing

### 3.1. RFDM

**Figure 1:**
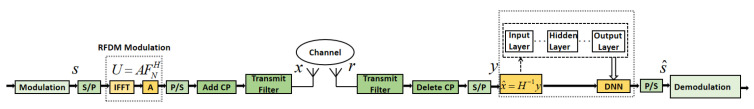
A RFDM-based multicarrier communication system.

The proposed RFDM-based single-input–single-output (SISO) communication architecture is constructed as shown in Figure 1. A message bit sequence is digitally modulated as $s\in C^{N\times 1}$ with the power constraint $\frac{1}{N}{∥s∥}^{2}=1$; the elements $s_{i},i=1,2,\ldots ,N$ are individually taken from constellation set Q, e.g., quadrature phase shift keying (QPSK) and quadrature amplitude modulation (QAM). Following serial-to-parallel (S/P) conversion and RFDM modulation, the obtained signal x is

(3) $$x={\mathrm{AF}}_{N}^{H}s=Us,$$

where $F_{N}$ denotes the *N*-point normalized FFT matrix and $A\in C^{M\times N}\left(M<N\right)$ denotes the sensing matrix of CS, in which we use Gaussian random matrices here. And $U={\mathrm{AF}}_{N}^{H}\in C^{M\times N}$ denotes the random frequency (RF) transform. Then, a CP of a length at least equal to the maximum channel delay spread is added to x. After the transmit filter, the signal x is sent out.

The received signal r[n] at the *n*-th slot is given by

(4) $$r\left[n\right]=\sum_{p=0}^{P-1}x\left[n-p\right]g\left[n,p\right]+v\left[n\right],$$

where

(5) $$g\left[n,p\right]=\sum_{i=1}^{L}h_{i}e^{j2\pi f_{i}\left(nT_{s}-pT_{s}\right)}P_{rc}\left(pT_{s}-\tau_{i}\right),$$

p=0,1,…,P−1, *P* is the channel tap, i=1,2,…,L, *L* is the number of multipaths, $T_{s}$ is the system sampling interval, $\left\{h_{i},f_{i},\tau_{i}\right\}$ represent the complex channel gain, the Doppler shift, and the delay at the *i*-th path. $P_{rc}$ is the raised-cosine roll-off filter which can reduce signal bandwidth and intersymbol interference.

### 3.2. DNN Detection Method

After the receiver filter and CP removal, (4) can be written as

(6) $$y=Hx+v={\mathrm{HAF}}_{N}^{H}s+v.$$

Since the matrix A can compress the signal which will require fewer subcarriers or the number of antenna for transmitting the same data, and the original s is not sparse, these lead to the challenge of RFDM: developing a low-complexity detector with acceptable accuracy. This detector consists of two parts: (1) Estimate $\hat{x}$ according to the channel information as $\hat{x}=H^{-1}y=x+H^{-1}v$. (2) Reconstruct the original signal $\hat{s}$ based on the DNN, as shown in Figure 1.

DNN, as one of the well-known algorithms of DL, can automatically learn and extract features from raw data, effectively model complex nonlinear relationships, and powerful computational capabilities to efficiently train on large datasets, achieving better generalization performance and enhancing the understanding and representation of data.

Generally, a DNN consists of *C* layers: an input layer, C−2 hidden layers, and an output layer. Given an input vector d, the output of every layer can be written as follows:

(7) $$d_{c}=f\left(W_{c}\cdot d_{c-1}+b_{c}\right),$$

where $d_{c-1}$ represents the input of the *c*-th layer, $d_{c}$ denotes its output, and $W_{c}$ is the weight matrix associated with the (c−1)-th and *c*-th layers, which can be continuously updated during the training process using the backpropagation algorithm to minimize the loss function, allowing the network to better adapt to the training data. The weight matrices of different layers can learn various levels of features, assisting the network in more complex pattern recognition. $b_{c}$ is the bias vector and f(·) is the activation function.

Consequently, the output of DNN can be mathematically expressed as:

(8) $$\hat{s}=F\left(\hat{x};\Theta \right)=f^{\left(C\right)}\left(f^{\left(C-1\right)}\left(\cdots f^{\left(1\right)}\left(\hat{x}\right)\right)\right),$$

where Θ represents the learned parametric set and $\hat{x}$ is the input of the network. The parameter set Θ is optimized by reducing the loss function defined as the distance between the prediction and the regression vector. We define *T* as the size of the dataset. The pair ${\left\{x^{\left(t\right)},s^{\left(t\right)}\right\}}_{t=1}^{T}$ is utilized to train Θ to minimize the loss function. The mean squared error (MSE) is adopted as the loss function to express the distance between the transmitted vector x and the output of DNN s as:

(9) $$L\left(x,s\right)=\sum_{t=1}^{T}{\left(x^{\left(t\right)}-{\mathrm{AF}}_{N}^{H}s^{\left(t\right)}\right)}^{2}.$$

Although increasing the number of hidden layers, neurons, and samples increases the training time, this DNN model can be trained offline in advance. Once the parameters Θ of DNN model are obtained, the testing process is very fast. Then, the DNN-based network can predict $\hat{s}$ from $\hat{x}$.

It is important to note that the dataset collection process and DNN training are performed without affecting the classical communication system operation. Hence, it is feasible to collect a large dataset for capturing the dynamics in the environment because it does not interfere with the classical system operation.

## 4. Rfdm-Mimo

**Figure 2:**
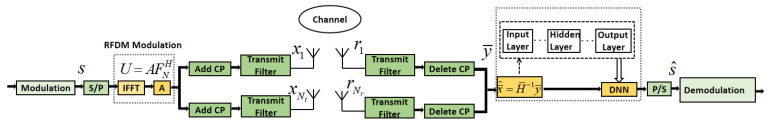
A RFDM-based multicarrier MIMO communication system.

Let us consider the RFDM scheme in a multi-antenna scenario; Figure 2 shows the RFDM-MIMO scheme with the number of transmitting antennas $N_{t}$ and receiving antennas $N_{r}$. Modulated signal s through S/P and RFDM modulation is divided into $N_{t}$ segments, and CP is added to each segment. After the transmit filter, signal $x_{j}$ is obtained and transmitted through the channel at the *j*-th antenna, $j=1,\ldots ,N_{t}$. After discarding the CP, the received signal $\bar{y}$ can be rewritten as:

(10) $$\bar{y}=\bar{H}\bar{x}+\bar{v},$$

where $\bar{y}={\left[y_{1}^{T},\ldots ,y_{N_{r}}^{T}\right]}^{T}\in C^{NN_{r}\times 1}$, $\bar{x}={\left[x_{1}^{T},\ldots ,x_{N_{t}}^{T}\right]}^{T}\in C^{NN_{t}\times 1}$, $\bar{H}={\left[H_{1}^{T},\ldots ,H_{N_{r}}^{T}\right]}^{T}\in C^{NN_{r}\times NN_{t}}$, $\bar{v}={\left[v_{1}^{T},\ldots ,v_{N_{r}}^{T}\right]}^{T}\in C^{NN_{r}\times 1}$.

At the receiver, the complete RFDM-MIMO detector is like this: (1) Estimate $\hat{\bar{x}}$ according to the channel information:

(11) $$\hat{\bar{x}}={\bar{H}}^{-1}\bar{y}=\bar{x}+{\bar{H}}^{-1}\bar{v}.$$

(2) Reconstruct the original signal $\hat{s}$ based on the pre-trained DNN, as shown in Figure 2.

## 5. Numerical Results

In this section, RFDM is compared to MMSE detectors in mobile time-varying channels. The multicarrier modulations use the same bandwidth, i.e., the system bandwidth is 1.4 MHz, the subcarrier spacing is Δf = 15,000 Hz, and the vehicle speed is 120 km/h with a maximum Doppler frequency shift 300 Hz. The number of subcarriers in the MMSE detector is $N_{s}=128$ and the compression ratio ρ=0.75, which means that the number of subcarriers for our RFDM is $N_{s}=96$. The maximum number of multipath delay channel taps L=9, and BPSK and QPSK are employed. Furthermore, the antenna number is considered with $N_{t}=N_{r}=4$. Many simulation setups are from the 3GPP LTE-OFDM system [14]. We also assume that the channel estimation is perfect at the receiver and so we run 50,000 Monte Carlo simulations to estimate the average BER.

The sample of the training set is 50,000 to train the DNN model offline. The hidden layer produces an output after weighting (default function ‘tansig’) the input and summing with the deviation (default function ‘learngdm’); the hidden layer produces an input to the output layer after activating the function (default function ‘Sigmoid’); and finally, the hidden layer produces an output after weighting the input and summing with the deviation and passing through the corresponding function (default function ‘purelin’). We set three layers—the input, hidden and output layers—in our simulations for simplicity. Once the *DNN* model is trained, then the receiving signal $\hat{x}$ is passed as the input to the *DNN* model to detect the original signal $\hat{s}$.

**Figure 3:**
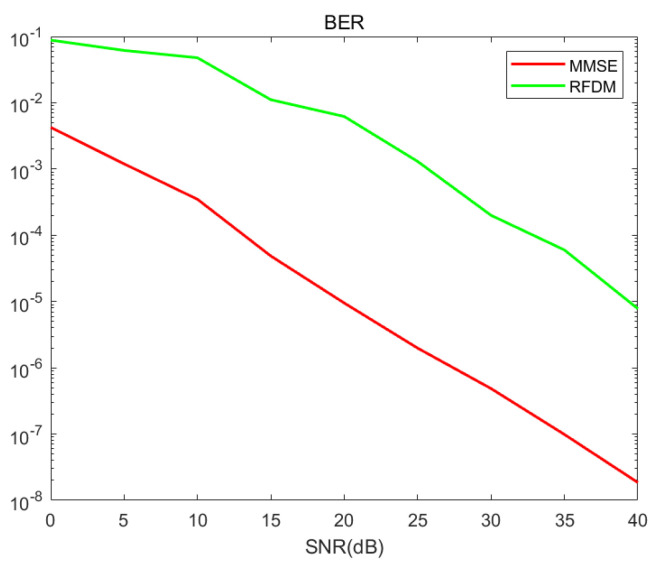
BER comparisons of RFDM with BPSK in 4 × 4 MIMO.

**Figure 4:**
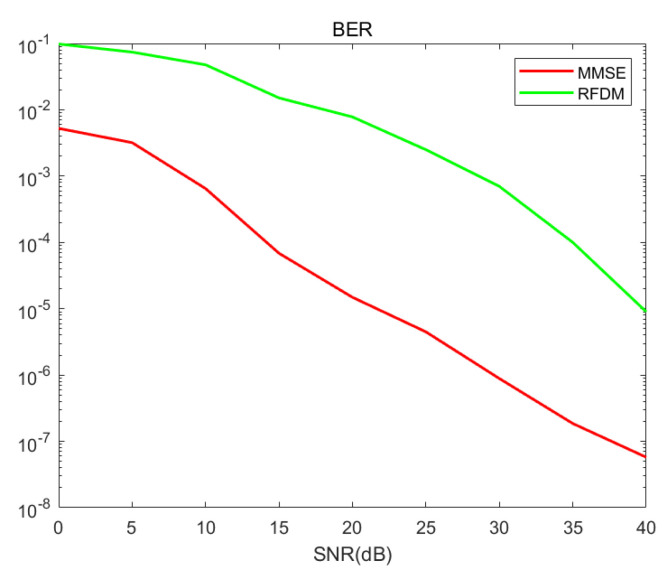
BER comparisons of RFDM with QPSK in 4 × 4 MIMO.

Figure 3 and Figure 4 present the BER performance of the proposed RFDM and the MMSE scheme when using BPSK and QPSK modulation, respectively. It shows that our solution currently has a performance gap with MMSE, but when SNR exceeds 20, our bit error rate can achieve $10^{-3}$ which is still competitive, especially since MMSE uses 128 subcarriers while we only use 96 subcarriers.

## 6. Conclusions

In this paper, we propose a novel RFDM scheme. Specifically, the signal is compressed to lower dimensions at the transmitter and can be reconstructed at the receiver. This means that we need fewer subcarriers or antennas to transmit the same amount of data. The DNN model is trained offline to learn the mapping function between an original signal. The BER performance of this proposed scheme is not better than the MMSE detection algorithm; however, we think it is a good attempt when the same quantity is transmitted, although only three quarters of the number of subcarriers of the MMSE scheme is used. It is still very important to improve the transmission speed. We will attempt to ascertain a better performance by avoiding the inversion of the channel matrix H, since its properties are directly considered and incorporated into DNN samples to train a good neural network model. There are too many training methods for neural network models, and ours used in this paper is too simple. We must optimize or find a better model to detect the signal. In addition, considering the properties of matrix A, the transmission scheme must be optimized comprehensively. 

## Data Availability

The data presented in this study are available on request from the corresponding author.
